# Editorial: Machine learning in radiation oncology

**DOI:** 10.3389/fonc.2022.1032858

**Published:** 2022-09-26

**Authors:** Wei Zhao, Ye Zhang, Jia Wu, Xiaomeng Li, Yuming Jiang

**Affiliations:** ^1^ Department of Physics, Beihang University, Beijing, China; ^2^ Beihang Hangzhou Innovation Institute, Yuhang Xixi Octagon City, Hangzhou, China; ^3^ Center for Proton Therapy, Paul Scherrer Institute (PSI), Villigen, Switzerland; ^4^ Department of Imaging Physics, University of Texas MD Anderson Cancer Center, Houston, TX, United States; ^5^ Department of Electronic and Computer Engineering, Hong Kong University of Science and Technology, Kowloon, Hong Kong SAR, China; ^6^ Department of Radiation Oncology, Stanford University, Stanford, CA, United States

**Keywords:** machine learning, deep learning, radiation oncology, radiomics, radiotherapy, image segmentation, artificial intelligence

Machine learning (ML) excels in learning complex relationships and incorporating existing prior knowledge into an inference model. The model is able to recognize complex patterns in medical data using either human-engineered features or deep neural network representations. Given the nature of heavy data involvement of ML and a large amount of existing routine data, such as computed tomography images, organs and targets contours, treatment plans, and prognosis records, radiation oncology is one of the best fields for ML algorithms. ML has the potential to benefit the whole workflow of radiotherapy: from patient modeling, image segmentation, treatment planning, patient setup, and prognosis analysis. Indeed, the Food and Drug Administration has cleared numerous ML-based technologies with regard to various radiation oncology aspects and there are having transformative ML-based applications in clinical practice.

The remarkable progress in ML-based radiation oncology has driven the special issue. Specifically, it consists of 13 papers that cover various aspects of radiation oncology. Liu et al. develop an experimental-computational approach to predict the changes in cell number over time in response to fractionated radiation. By developing a mathematical model using time-resolved microscopy, together with phase contrast images and Cytotox Red images, this study establishes a framework to quantitative characterize and predict the dynamic radiobiological response of 9L and C6 glioma cells to fractionated radiotherapy for the first time.

Because it is challenging to differentiate Germinomas of the basal ganglia from gliomas by using routine MRI images. Ye et al. use transfer learning to fine-tune a pre-trained ResNet18 to identify germinomas from gliomas with manually segmented MRI images acquired using T1C sequence. A mean area-under-curve (AUC) of 0.88 is achieved, showing the accuracy of the model. This study has the potential to provide valuable pretreatment information. Ren et al. demonstrate the feasibility of generating lung perfusion images based on CT images for lung cancer patients. To this end, CT images of non-cancer patients are employed to synthesize perfusion images using a convolutional neural network (CNN), and the features learned from this process are then adapted specifically for lung cancer patients using a transfer learning framework. The synthetic perfusion images are compared with SPECT perfusion images which are generally considered to be the gold standard and show strong voxel-wise correlation and function-wise similarity. This study, therefore, suggests deep learning (DL) method is able to provide regional-based functional information for lung avoidance radiation therapy.

Typical treatment planning procedure in radiotherapy requires accurate target contouring, organs-at-risk (OARs) delineation, as well as iterative dose optimization. This procedure involves various steps and synergic work from different radiation therapy teams, such as radiation oncologists, medical physicists, and dosimetrists, and it is usually time-consuming and also suffered from inconsistency between team members. To reduce the workload of radiation oncologists for OARs delineation, Zhang et al. use a modified DenseNet to automatically delineate OARs in thoracic CT images. By subjecting the delineated OARs to treatment planning, the dosimetric parameters of the optimized dose show no statistically significant difference between autosegmentation and manual segmentation. In addition to OARs delineation, ML algorithms are also subjected to tumor volume segmentation. Tian et al. develop a semisupervised transfer learning method to segment glioblastoma on pretreatment MRI and the accuracy of the independent testing data has shown to be sufficient for radiotherapy treatment.

The DL-based autosegmentation can not only be employed for radiotherapy, but also for ablation therapy. Anderson et al. demonstrate fully convolutional neural networks provide rapid and accurate identification and segmentation of colorectal liver metastasis and ablation zones on contrast-enhanced CT scans, with positive physician reviews. The DL-based autosegmentation is also compared to classical atlas-based autosegmentation. Wang et al. evaluate the difference between atlas-based and DL-based autosegmentation of OARs for nasopharyngeal carcinoma patients. The study shows a well-trained DL segmentation model significantly outperforms atlas-based segmentation for nasopharyngeal carcinoma, especially for the OARs with small volumes.

A study further shows segmentation and dose optimization can be performed together. Cui et al. propose to accelerate the treatment planning procedure by introducing a DL-based CT-only dose prediction framework. This framework is able to simultaneously generate tumor masks and optimized doses by using a multi-task loss learning strategy. It can therefore significantly shorten the treatment planning procedure.

DL model performance relies on the distribution of the training data. Peng et al. show a homogeneous training dataset results in better performance than a non-homogeneous for dose prediction. Therefore, a patient-specific model trained using a consistent dataset is recommended for dose prediction. In case a homogeneous dataset is not available, additional beam information (i.e., beam mask) can improve the model performance to yield acceptable results.

ML algorithms are also popular in treatment outcome prediction. Kawahara et al. predict the local response of metastatic brain tumor to Gamma knife radiosurgery (GKRS) by using a neural network model with input radiomics features. The accuracy and sensitivity of the predictive model have been shown to outperform the traditional method. This study is able to help physicians to gain the most desirable treatment outcome for patients treated with GKRS. With regards to the adverse radiation effects (ARE) of stereotactic radiotherapy (SRT) in the treatment of brain metastases (BM) in solid cancers, Keek et al. train models based on radiomics features, DL features, and patient characteristics to predict ARE risk before SRT. This is a great decision-making support tool to allow physicians to opt for the optimal treatment solution for patients with BM.

Yu et al. use ML models based on multiphasic CT radiomics features to differentiate benign and malignant parotid tumors. Qin et al. review the applications of radiomics in radiation oncology with a focus on locally advanced rectal cancer. This paper also summarizes the research of dosiomics which is an emerging Research Topic that involves dose-based radiomics analysis in radiation treatment.

Within the spectrum of ML applications in radiation oncology, image segmentation plays a central role. It has attracted the most research and translational activities and resulted in five papers in this Research Topic. While DL-based autosegmentation is able to reduce the workload of physicians and improve work efficiency, its accuracy still needs further justification. Radiomics-based applications including outcome prediction and ARE are also under extensive investigation. DL features used together with conventional radiomics features is the new frontier and usually yield better model prediction. As a core section of radiotherapy, dose prediction is a hot topic and results in two papers. Overall, the progress of ML in radiation oncology has been well reflected by this special collection, with major progress in diagnosis, image segmentation, and radiomics well covered. We anticipate ML applications will continue to play an important role in radiation oncology and translational work will be performed to improve clinical efficiency.

## Author contributions

WZ and XL performed the analysis of the collection and wrote the manuscript. YZ, JW, and YJ helped with the analysis and study design. YZ and JW devised the project’s aim and supervised the progression of the project. All authors contributed to the article and approved the submitted version.

## Funding

This work was supported in part by the National Natural Science Foundation of China (No. 12175012).

## Conflict of interest

The authors declare that the research was conducted in the absence of any commercial or financial relationships that could be construed as a potential conflict of interest.

## Publisher’s note

All claims expressed in this article are solely those of the authors and do not necessarily represent those of their affiliated organizations, or those of the publisher, the editors and the reviewers. Any product that may be evaluated in this article, or claim that may be made by its manufacturer, is not guaranteed or endorsed by the publisher.

